# Influence of corneal spherical aberration on prediction error of the Haigis-L formula

**DOI:** 10.1038/s41598-020-63594-4

**Published:** 2020-04-15

**Authors:** Woong-Joo Whang, Young-Sik Yoo, Hyun-Seung Kim, Geunyoung Yoon

**Affiliations:** 1 Department of Ophthalmology, Yeouido St. Mary’s Hospital, College of Medicine, The Catholic University of Korea, Seoul, Korea; 2 Department Ophthalmology, Uijeongbu St. Mary Hospital, College of Medicine, The Catholic University of Korea, Uijeongbu, Korea; 3 Department of Ophthalmology, Seoul St. Mary’s Hospital, College of Medicine, The Catholic University of Korea, Seoul, Korea; 4 Flaum Eye Institute, Center for Visual Science, The Institute of Optics, University of Rochester, Rochester, New York USA

**Keywords:** Lens diseases, Outcomes research

## Abstract

The purpose of this study is to investigate the relationships between corneal asphericity and Haigis-L formula prediction errors in routine cataract surgery after refractive surgery for myopic correction. This retrospective study included 102 patients (102 eyes) with a history of previous PRK or LASIK and cataract surgery. Axial length, anterior chamber depth, and central corneal power were measured using the optical biometer. On the anterior corneal surface, Q-value, spherical aberration, and ecentricity at 6.0 and 8.0 mm were measured using a rotating Scheimpflug camera. The postoperative refractive outcome at 6 months, mean error, and mean absolute error were determined. Correlation tests were performed to determine the associations between pre-cataract surgery data and the prediction error. The Q-values for 6.0 and 8.0 mm corneal diameter were 1.57 ± 0.70 (range: 0.03~3.44), and 0.82 ± 0.5 (range: −0.10~−2.66). The spherical aberration for 6.0 and 8.0 mm diameter was 1.16 ± 0.39 µm (range: 0.24~2.08 µm), and 3.69 ± 0.87 µm (range: 0.91~5.91 µm). eccentricity for 6.0 and 8.0 mm diameter was −1.22 ± 0.31 (range: −1.85 to −0.17), and −0.82 ± 0.39 (range: −1.63 to 0.32). The spherical aberration for 8.0 mm cornea diameter showed the highest correlations with the predicion error (r = 0.750; p < 0.001). When the modified Haigis-L formula considering spherical aberration for 8.0 mm produced smaller values in standard deviation of mean error (0.45D versus 0.68D), mean absolute error (0.35D versus 0.55D), and median absolute error (0.31D versus 0.51D) than the Haigis formula. Corneal asphericity influences the predictive accuracy of the Haigis-L formula. The accuracy was enhanced by taking into consideration the corneal spherical aberration for the 8.0 mm zone at pre-cataract surgery state.

## Introduction

As the number of laser refractive surgeries for myopia increases, phacoemulsification combined with IOL implantation after refractive surgery has become common. However, performing cataract surgery after corneal refractive surgery is more challenging than on the normal cornea without any surgical history; this is because patients with history of refractive surgery have higher expectations and the calculation of appropriate IOL power is more difficult since most of the conventional equations used for IOL power calculation have been developed on the basis of the postoperative refraction of cataract surgery on eyes without any history of corneal refractive surgery^1^. Nevertheless, numerous methods and formulas have been introduced, and the predictive accuracy of IOL power calculation formulas used after corneal refractive surgery has been increased. However, their predictability is still lower than that of an IOL power calculation for a normal cornea and does not meet the standards of the British National Health Service published in 2009^2,3^. They proposed that 55% of cataract surgeries be performed within 0.5 diopters (D) and 85% be within 1.0 D from the targeted spherical equivalent^2^.

The Haigis-L formula accurately predicted the postoperative refractive outcomes in eyes with a previous myopic excimer laser history^4,5^ and shows better predictability than any other no-history method^3^. It corrects both the corneal radius measurement error and keratometric refractive index error by using a regression equation. The equation was derived by correlating post-refractive surgery corneal radii measured using the IOLMaster partial coherence interferometer with actual effective corneal powers calculated using the refractive history method. It additionally subtracts 0.35 D from the effective corneal power to eliminate prediction error from the estimated lens position. Finally, the corrected corneal radius is entered into the regular Haigis formula to calculate IOL power. Meanwhile, the Haigis-L formula has a limitation. Although it considers the anterior-posterior corneal power relationship changes after excimer laser surgery and corrects the IOLMaster measurement of the corneal radius^4^, both the ablation depth and ablation volume for myopic laser vision correction are different for each individual and the differences are not considered in the calculation process of the Haigis-L formula.

The corneal shape assumes a more oblate optical architecture after myopic PRK (Photorefractive keratectomy) or LASIK (Laser *in situ* keratomileusis), and the amount of induced asphericity increases with the amount of myopic correction during surgery^6–9^. Savini *et al*.^10^ concluded that the asphericity on the anterior cornea influenced the refractive outcomes of IOL power calculations predicted by the Haigis, Hoffer Q, Holladay 1, and SRK/T formulas in normal corneas. Another study concluded that corneal asphericity on the anterior surface influenced the refractive outcomes calculated by the SRK/T formula in the eyes with a prior myopic LASIK history^11^. Considering corneal spherical aberration also improved the predictability of refractive outcomes in post-LASIK eyes when the ray tracing prediction was applied^12^.

The purpose of this study was to investigate the relationships between pre-cataract surgery corneal spherical aberration induced by the anterior corneal surface, and the prediction errors in the refractive outcomes derived from the Haigis-L formula. We also aimed to modify the Haigis-L formula according to the correlation between biometric measurements and the prediction error and to enhance the predictive accuracy.

## Methods

This retrospective study included 102 eyes of 102 patients who underwent cataract surgery at Yeouido St. Mary Hospital (Seoul, Korea) between July 2017 and March 2019. All 102 patients had a history of previous PRK or LASIK for myopia correction. Total 102 eyes were divided into a training set (80 eyes with cataract surgery from July 2017 to Sep 2018) and a test set (22 eyes with cataract surgery from Oct 2018 to March 2019). Verbal informed consent was obtained from all patients, and the study methods adhered to the tenets of the Declaration of Helsinki for the inclusion of human participants in biomedical research. This study was approved by the Institutional Review Board (IRB) of Yeouido St. Mary Hospital. All patients who underwent myopic refractive surgery and who had their IOL power calculated using the Haigis-L formula were included in this study. No patient had a history of ocular disease, general disorders affecting the cornea, previous ocular surgery except PRK or LASIK, and none had intraoperative complications.

The axial length, corneal radius, and anterior chamber depth were provided from the IOLMaster optical biometer (version 5; Carl-Zeiss, Germany). The IOLMaster uses an infrared laser (780 nm) to measure the axial length using the principle of partial coherence interferometry and slit projection techniques to assess the anterior chamber depth. 6 light spots hexagonally projected in a 2.3 to 2.5 mm radius onto the cornea record the reflections by measuring the separation between opposite pairs of light spots, and calculates toroidal surface curvature^13^. Both the Q-value and the spherical aberration on the anterior corneal surface were measured using a rotating Scheimpflug camera to assess corneal asphericity. The spherical aberrations were calculated from the Zernike analysis map in the anterior corneal surface and measured at the 6.0 mm zone and 8.0 mm zone. A Pentacam HR (Oculus, Wetzlar, Germany) analyzed the cornea via a 25 image scan, and only those scans that had an examination quality specification graded by the instrument as “OK” were included in this study.

All Surgeries was performed through a self-sealing, 2.2 mm temporal clear corneal incision under topical anesthesia. Phacoemulsification was performed using the Centurion System (Alcon, USA). Following phacoemulsification, 1 kind of IOL (Tecnis ZCB00, Abbott Medical Optics Inc., Santa Ana, USA) was inserted into the capsular bag in all eyes.

The optimized IOL constants published on the ULIB (User Group for Laser Interference Biometry) website (http://www.augenklinik.uni-wuerzburg.de/ulib/c1.htm) were applied for IOL power calculation. The IOL constants of ZCB00 IOL were − 1.302 (a0), 0.210 (a1), and 0.251 (a2). The targeted refraction and IOL power for the Barrett True-K, and Shammas-PL formulas were calculated using the American Society of Cataract and Refractive Surgery IOL power calculator, version 4.0 (http://iol.ascrs.org/) The postoperative refractive outcome was measured 6 months postoperatively by subjective refraction.

The Haigis-L formula was modified by the regression equation and the following thin-lens formula.

$$\mathrm{IOL}\,\mathrm{power}=\frac{1336}{{AL}-{ELP}}-\frac{1336}{\frac{1336}{Z}-{ELP}}$$

Z = Effective equivalent corneal power

$${\rm{Effective}}\,{\rm{equivalent}}\,{\rm{corneal}}\,{\rm{power}}=-\,5.6125\,\times \,r+82.2603-0.35$$

AL:axial length; ELP:effective lens position; r:corneal radius

The prediction error was the actual postoperative spherical equivalent minus the predicted SE, The mean error (ME) was the average value of prediction errors and the mean absolute error (MAE) was the average absolute value of the prediction errors. The median error was a median value of prediction errors, and the median absolute error as a median value of absolute prediction errors. The percentages of refraction prediction within ± 0.50 D, ± 1.00 D, and ± 1.50 D were obtained.

Pearson’s correlation analyses and linear regression analyses were performed to determine the relationships between biometry at pre-cataract surgery state (axial length, anterior chamber depth, anterior corneal radius, Q-value, spherical aberration, and eccentricity) and the prediction obtained using the Haigis-L formula. For the comparison between a training set and a test set, the Chi-square test and the Mann-Whitney test were used. They were also used for the comparison according to the timing of PRK or LASIK in a training set. The statistical analyses were performed using IBM SPSS Statistics for Windows (Version 21.0; IBM Corp., Armonk, USA), and the level of significance was set at 0.05.

## Results

IOLMaster biometry, Pentacam rotating Scheimpflug imaging, and stable manifest refraction were all available for 102 eyes. The mean spherical equivalents were −5.32 ± 5.47 D preoperatively and −1.59 ± 1.43 D at 6 months after cataract surgery in a training set. In a test set, the mean spherical equivalents were −4.97 ± 3.24 D preoperatively and −1.14 ± 1.34 D at 6 months after cataract surgery. Table 1 shows demographic data in a training set and a test set. There was no difference in axial length, anterior chamber depth, mean corneal radius, corneal asphericity, PRK:LASIK ratio, and period time after PRK/LASIK (all *p* > 0.05) between a training set and a test set.

**Table 1:** Demographic data in a training set (n = 80) and a test set (n = 22).

	training set	test set	*P* value
Eyes	80	22	
F:M	50:30	14:8	*0.92
Age	51.67 ± 9.95 (range: 33 to 68)	52.18 ± 7.62 (range: 39 to 63)	**0.96
Axial length (mm)	28.43 ± 2.29 (range: 24.47 to 33.45)	27.58 ± 1.72 (range: 24.64 to 31.09)	**0.11
Anterior chamber depth (mm)	3.62 ± 0.37 (range: 2.28 to 4.73)	3.72 ± 0.33 (range: 2.69 to 4.51)	**0.41
Mean anterior corneal radius (mm)	8.79 ± 0.53 (range: 7.82 to 10.28)	8.66 ± 0.38 (range: 8.06 to 9.36)	**0.38
Q-value at 6.0 mm	1.57 ± 0.70 (range: 0.03 to 3.44)	1.48 ± 0.76 (range: 0.42 to 3.67)	**0.41
Q-value at 8.0 mm	0.82 ± 0.58 (range: −0.10 to 2.66)	0.71 ± 0.49 (range: −0.03 to 1.84)	**0.40
Spherical aberration at 6.0 mm (µm)	1.16 ± 0.39 (range: 0.24 to 2.08)	1.16 ± 0.29 (range: 0.25 to 1.66)	**0.75
Spherical aberration at 8.0 mm (µm)	3.69 ± 0.87 (range: 1.20 to 5.49)	3.62 ± 0.53 (range: 2.95 to 5.16)	**0.36
Eccentricity at 6.0 mm	−1.22 ± 0.31 (range: −1.85 to −0.17)	−1.18 ± 0.30 (range: −1.92 to −0.65)	**0.42
Eccentricity at 8.0 mm	−0.82 ± 0.39 (range: −1.63 to 0.32)	−0.78 ± 0.34 (range: −1.36 to 0.17)	**0.41
IOL power (diopter)	19.01 ± 4.26 (range: 6.0 to 26.5)	19.39 ± 2.66 (range: 14.0 to 24.0)	**0.23
PRK: LASIK ratio	28:52	7:15	*0.78
Period time after PRK / LASIK (year)	13.79 ± 5.25 (3 to 26)	13.00 ± 5.44 (4 to 24)	**0.52

*P value by chi-square test

**P value by the Mann-Whitney U test.

**Figure 1:**
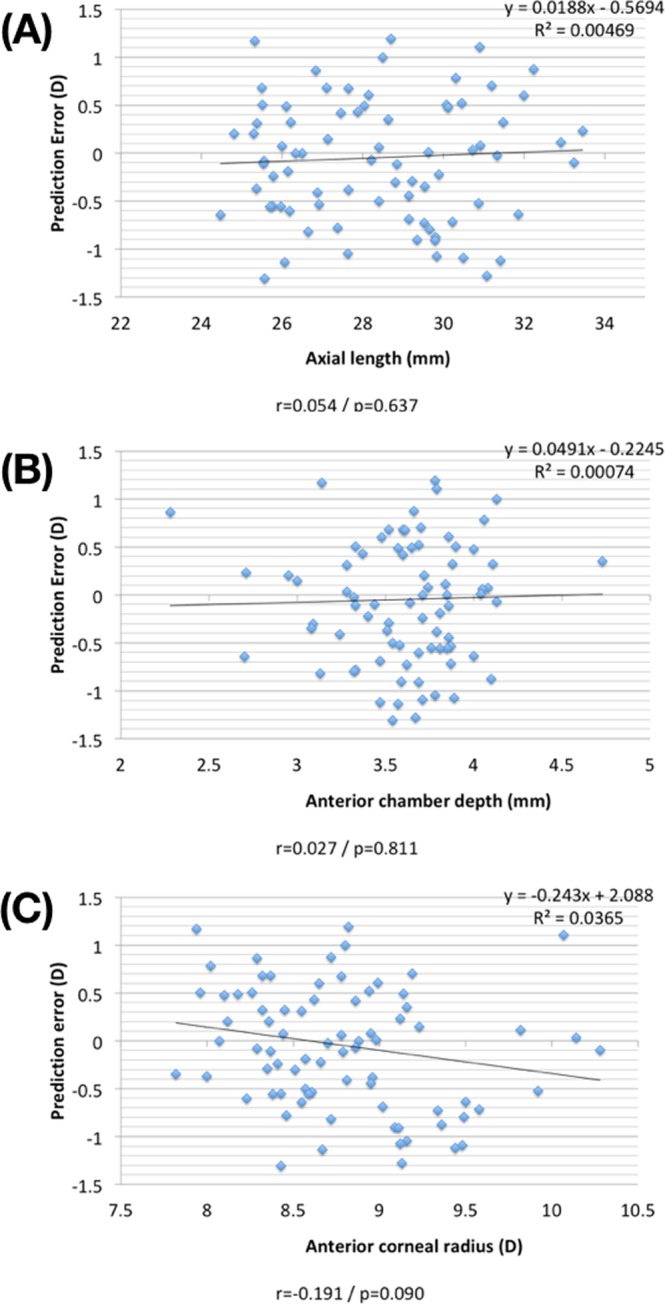
The biometric measurements before cataract surgery obtained using the IOLMaster and the prediction error (postoperative refraction – predicted refraction) calculated using the Haigis-L formula are shown on scatter plots. (**A**) Axial length versus the prediction error, (**B**) anterior chamber depth versus the prediction error and (**C**) anterior corneal radius versus the prediction error.

Figure 1 shows the relationships between preoperative biometric data from partial coherence interferometry and the prediction error derived from the Haigis-L formula. The axial length, anterior chamber depth, and anterior corneal radius were not statistically correlated with mean error (r = 0.054, 0.027, −0.191; p = 0.637, 0.811, and 0.090, respectively).

**Figure 2:**
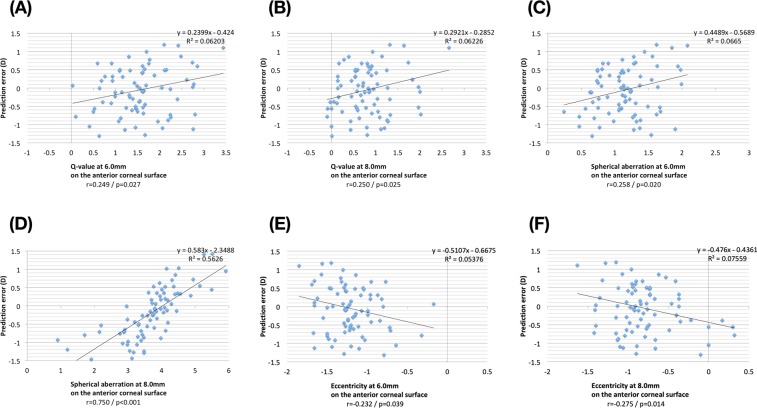
The corneal asphericity before cataract surgery determined using the Pentacam Scheimpflug camera and the prediction error (postoperative refraction – predicted refraction) calculated using the Haigis-L formula are shown on scatter plots. (**A**) Q-value at 6.0 mm versus the prediction error, (**B**) Q-value at 8.0 mm versus the prediction error, (**C**) spherical aberration at 6.0 mm versus the prediction error, (**D**) spherical aberration at 8.0 mm versus the prediction error, (**E**) eccentricity at 6.0 mm versus the prediction error, and (**F**) eccentricity at 8.0 mm versus the prediction error.

Figure 2 illustrates the association between corneal asphericity and prediction error. The Q-values at 6.0 mm and 8.0 mm showed positive correlations with the prediction error derived from the Haigis-L formula. (r = 0.249, and 0.250; p = 0.027 and 0.025, respectively) The spherical aberrations at 6.0 mm and 8.0 mm also showed positive correlations with the mean error (r = 0.258, and 0.750; p = 0.020 and <0.001, respectively). The eccentricity at 6.0 mm and 8.0 mm showed negative correlations with the prediction error derived from the Haigis-L formula. (r = −0.232, and −0.275; p = 0.039 and 0.014, respectively) In particular, the spherical aberration at 8.0 mm showed the highest correlation, and the regression equation was as follows:

$$PE=0.583\cdot S{A}_{8mm}-2.3488,$$

***PE***: the prediction error derived from the Haigis-L formula, **SA**_**8mm**_: the spherical aberration of the anterior corneal surface at 8.0 mm as determined by a rotating Scheimpflug camera

Presently, the modification of the Haigis-L formula according to the spherical aberration at 8.0 mm would be crucial for accurate prediction of IOL power.

We corrected effective equivalent corneal power of the Haigis-L formula considering the influence of corneal spherical aberration at 8.0 mm zone by transforming data from the spectacle plane (VD = 12.0 mm) to corneal plane.

The modified Haigis-L formula calculated by the regression formula in this study is as follows:

$$IOL\,power=\frac{1336}{AL-ELP}-\frac{1336}{\frac{1336}{Z{\prime} }-ELP}$$

PE = 0.583  $$\times $$  SA_8mm_ − 2.3488

$$REFc=\frac{1000\,\times REFs}{1000-REFs\times VD}$$

Z′ (New effective equivalent corneal power considering SA)

$$=\,(\,-\,5.6125r+82.2603\,\mbox{--}\,0.35)-\frac{1000\,\times \,(0.583\times {\rm{SA}}-2.3488)}{1000-(0.583\times {\rm{SA}}-2.3488)\,\times \,VD}$$

AL:axial length; ELP:effective lens position; r:corneal radius; REFc:dioptric power on corneal plane; REFs:dioptric power on spectacle plane; SA:spherical aberration at 8.0 mm; VD:vertex distance.

Table 2 shows the mean error, median error, mean absolute error, median absolute error, and percentages of refraction prediction within ± 0.50 D, ± 1.00 D, and ±1.50 D for all 80 eyes. Comparing with the Haigis-L formula, the modified Haigis-L formula produced smaller values in standard deviation of mean error (0.45D versus 0.68D), median absolute error (0.31D versus 0.51D), and narrower interquartile range of prediction error (0.63D versus 1.04D).

**Table 2:** Mean error and mean absolute error calculated using the Haigis-L formula and the modified Haiigs-L formula considering spherical aberration at 8.0 mm in 80 eyes after refractive surgery and refraction prediction within ± 0.50 D, ± 1.00 D, and ± 1.50 D.

	Haigis-L formula	Modified Haigis-L formula
Eyes	80	80
ME (D) ± SD	−0.05 ± 0.68 (range: − 1.31 to 1.57)	0.00 ± 0.45 (range: − 0.96 to 0.97)
Median error (D)	−0.03	−0.02
MAE (D) ± SD	0.55 ± 0.38 (range: 0 to 1.57)	0.35 ± 0.27 (range: 0 to 0.97)
Median absolute error (D)	0.51	0.33
Interquartile range (D)	1.04	0.63
Within 0.5 D (%)	47.5	70.0
Within 1.0 D (%)	85.0	100.0
Within 1.5 D (%)	97.5	100.0

D: diopters

ME: mean error

MAE: mean absolute error

SD: standard deviation.

Comparison with existing IOL formulas in a test set is listed in Table 3. Modified Haigis-L formula yielded the smallest median absolute error (0.19D) and narrowest interquartile range of prediction error (0.46D).

**Table 3:** Mean error and mean absolute error form the modified Haigis-L formula in 22 eyes after refractive surgery and refraction prediction within ± 0.50 D, ± 1.00 D, and ± 1.50 D. The predictive accuracy of the modified Haigis-L formula.

	Haigis-L formula	Modified Haigis-L formula	Barret True-K	Shammas-PL
Eyes	22	22	22	22
ME (D) ± SD	−0.18 ± 0.72 (range: −1.34 to 1.93)	0.06 ± 0.59 (range: −0.93 to 1.27)	0.20 ± 0.68 (range: −0.59 to 2.16)	0.15 ± 0.68 (range: −0.66 to 2.30)
Median error (D)	−0.27	0.04	0.13	0.09
MAE (D) ± SD	0.56 ± 0.48 (range: 0 to 1.93)	0.41 ± 0.41 (range: 0 to 1.27)	0.53 ± 0.46 (range: 0 to 2.16)	0.47 ± 0.50 (range: 0 to 2.30)
Median absolute error (D)	0.41	0.19	0.40	0.31
Interquartile range (D)	0.86	0.46	0.92	0.60
Within 0.5 D (%)	54.5	63.6	59.1	63.6
Within 1.0 D (%)	90.9	90.9	90.9	90.9
Within 1.5 D (%)	95.5	100.0	95.5	95.5

D: diopters

ME: mean error

MAE: mean absolute error

SD: standard deviation.

## Discussion

In this study, we found that the prediction error derived from the Haigis-L formula increased with an increase in corneal asphericity. This indicates that a more oblate cornea with larger amount of myopic excimer laser vision correction produces a more hyperopic result, whereas a relatively less oblate cornea with smaller amount of correction produces a more myopic result. To our knowledge, this is the first study investigating the influence of corneal asphericity in the Haigis-L formula.

Third-generation formulas aim to predict the IOL position more accurately by incorporating the effect of corneal curvature^14^. In particular, because the SRK/T formula shows high predictability in highly myopic eyes^15^, it has been widely used in cases with a previous history of refractive surgery for myopia. When data collected prior to the refractive surgery are available, IOL power can be calculated using the double-K clinical history method^16,17^. It uses 2 K-values: the pre-refractive surgery K for the calculation of effective lens position(ELP) and the post- refractive surgery K for vergence formula. If there is no previous refractive surgery history, Savini G *et al*.^18^ introduced a new method predicting the pre-refractive surgery anterior corneal radius and ELP from the posterior corneal radius and posterior Q-value at the state of post-refractive surgery. The Q-value on the posterior corneal surface was applied to eliminate ELP prediction error, not to reduce the radius and keratometric index error.

In contrast, the Haigis formula^19^ and the Haigis-L formula^4^, which are both fourth-generation formulas, do not consider preoperative corneal power and instead use preoperative anterior chamber depth for effective lens position prediction. Consequently, in IOL calculations, they have the advantage of avoiding formula errors resulting from former refractive surgery^4^ and do not need the application of the double-K method.

The Haigis-L formula still shows good predictability in cataract surgery after refractive surgery for myopic correction. A previous study showed that the formula could accurately predict IOL power in Asian patients with little variation in accuracy over a range of axial lengths^5^. Moreover, it is one of the most accurate methods to predict IOL power and is less affected by axial length than are other methods requiring a clinical history^20^. Similarly, we found that the prediction error predicted by the Haigis-L formula was not correlated with preoperative axial length.

The IOLMaster measures central corneal power on the anterior corneal surface and extrapolates posterior corneal power by assuming a fixed ratio between the anterior and posterior curvatures and a fixed refractive index. However, this assumption does not hold for the eyes that have undergone previous corneal refractive surgery, which alters the anterior corneal curvature and induces subtle changes on the posterior corneal surface. As the central corneal tissue is ablated, the difference in corneal thickness between the central area and peripheral area increases. Consequently, the ratio of posterior to anterior radius of the corneal curvature increases and the equivalent refractive index decreases.

Haigis^4^ had introduced the concept of corrected corneal radius calculated by a regression formula. However, the change according to the amount of myopic correction was not taken into consideration. Even if the corneas have the same anterior corneal power, the corneal power before excimer laser surgery or the ablation depth could be different. Prior studies^21–23^ have also demonstrated that the postoperative equivalent index of refraction decreased with the amount of refractive correction. Consequently, myopic laser vision correction results in an overestimation of corneal power, i.e., an underestimation of IOL power. This trend is strengthened by higher amounts of myopic laser refractive correction. Nevertheless, the Haigis-L formula has the disadvantage of neglecting corneal asphericity and equivalent refractive index.

In this study, the spherical aberration at 8.0 mm showed a higher association with prediction error derived from the Haigis-L formula than did the spherical aberration at 6.0 mm, Q-values, and eccentricity. When cataract surgeons select the type of aspheric IOL to compensate for the positive spherical aberration of the cornea and to enhance optical quality, spherical aberration at 6.0 mm should be considered. However, our purpose was not to evaluate optical quality but to predict the amount of correction. In general, the excimer laser surgery for myopic correction has an optical zone of 6.0~7.0 mm, as well as a transitional zone. Moreover, corneal myopic refractive surgery preserving the preoperative corneal asphericity might be desirable^24^. Wavefront-optimized ablation profiles^25^, Q-factor-customized ablation profiles^26^, and wavefront-guided treatments^27^ have been introduced for preserving or eliminating already existing aberrations. Therefore, evaluating the spherical aberration in a smaller zone would inhibit the analysis of anatomical changes after excimer laser surgery.

We additionally divided a total 80 eyes into 2 subgroups according to the timing of PRK or LASIK. Set July 2004 (approximately 15 years before cataract surgery) as the border, a total of 80 eyes are divided into two groups. We analyzed 38 cases of refractive surgery before July 2004 and 42 cases of PRK or LASIK afterwards. Table 4 shows comparisons between two groups and there was no difference in axial length, anterior chamber depth, corneal radius and Q-value/eccentricity or spherical aberration at 8.0 mm. However, significant difference in spherical aberration at 6.0 mm was revealed. These results suggest that the spherical aberration at 6.0 mm is affected by the ablation profile method, but corneal asphericity (Q-value and spherical aberration) at 8.0 mm are parameters that could predict ablation volume or ablation depth relatively independently of ablation profile.

**Table 4:** Demographic data clustered according to the timing of performing PRK or LASIK.

	Before July 2004	Since Aug 2004	*P* value
Eyes	38	42	
F:M	24:14	26:16	0.91*
Age	56.66 ± 9.74 (range: 33 to 68)	47.14 ± 7.82 (range: 34 to 58)	<0.001**
Axial length (mm)	28.35 ± 2.38 (range: 25.29 to 33.45)	28.50 ± 2.23 (range: 24.47 to 33.23)	0.72**
Anterior chamber depth (mm)	3.60 ± 0.37 (range: 2.28 to 4.13)	3.63 ± 0.38 (range: 2.70 to 4.73)	0.97**
Mean anterior corneal radius (mm)	8.79 ± 0.52 (range: 7.94 to 10.14)	8.78 ± 0.55 (range: 7.82 to 10.28)	0.81**
Q-value at 6.0 mm	1.68 ± 0.66 (range: 0.41 to 2.85)	1.48 ± 0.74 (range: 0.03 to 3.44)	0.28**
Q-value at 8.0 mm	0.80 ± 0.51 (range: −0.03 to 1.97)	0.83 ± 0.64 (range: −0.10 to 2.66)	0.98**
Spherical aberration at 6.0 mm (µm)	1.28 ± 0.40 (range: 0.47 to 2.08)	1.05 ± 0.34 (range: 0.24 to 1.85)	0.006**
Spherical aberration at 8.0 mm (µm)	3.67 ± 0.81 (range: 1.20 to 5.49)	3.71 ± 0.93 (range: 0.91 to 5.91)	0.99**
Eccentricity at 6.0 mm	−1.27 ± 0.26 (range: −1.69 to −0.64)	−1.17 ± 0.34 (range: −1.85 to −0.17)	0.28**
Eccentricity at 8.0 mm	−0.82 ± 0.36 (range: −1.40 to 0.17)	−0.82 ± 0.42 (range: −1.63 to 0.32)	0.98**
PRK: LASIK ratio	14: 24	14: 28	0.74*
Period time after PRK / LASIK (year)	18.39 ± 2.94 (15 to 26)	3.71 ± 0.93 (3 to 14)	<0.001**

*P value by chi-square test

**P value by the Mann-Whitney U test.

Traditionally, the clinical history method based on the information of prior excimer laser surgery, including the amount of myopic correction, has been regarded as the gold standard for calculating IOL power. However, historical data on corneal power and spherical equivalence measured near the time of corneal refractive surgery may not be available. Recent studies have proposed parameters associated with the amount of myopic correction. Schuster *et al*.^28^ concluded that the MEs predicted by the Holladay 1 and SRK/T formulas correlated with the ratio of posterior-to-anterior radius of corneal curvatures. They assumed that the posterior corneal surface does not change after myopic laser vision correction and hypothesized that the combination of a treated anterior surface and an unchanged posterior radius would reflect the amount of refractive treatment. The increase in oblateness was highly correlated with the attempted correction and the changes in corneal asphericity are good indicators reflecting the amount of myopic correction^6,8,9^. Canovas *et al*.^12^ demonstrated that the incorporation of corneal spherical aberration at 4.0 mm was important for improving accuracy in prediction of IOL power. Mori *et al*.^11^ concluded that both Q-value and spherical aberration showed statistically significant correlations with refractive error. However, they applied the formula designed for normal eyes and evaluated the Q-value only at 6.0 mm.

In this study, we also evaluated the Q-value and eccenricity. Although, the both parameters showed significant correlations with prediction error, the correlations were smaller than the spherical aberrations at both of 2 measurement zone.

Pentacam rotating Scheimpflug imaging was performed for assessing corneal asphericity. This is a noninvasive tool for examining the anterior segment^29^, and has an advantage of \ evaluating the entire anterior eye segment from the anterior corneal surface to the posterior lens surface in a single scan without any corneal contact. It provides complete corneal pachymetry and corneal topography within 2 s^30^. Prior studies^10,11,30^ have also evaluated the corneal asphericity at 6.0~8.0 mm by using the rotating Scheimpflug camera. Unlike the combined corneal topographer, the Scheimpflug camera measures the whole cornea in the same manner. Therefore, comparing the correlation coefficients for particular areas will not affect the reliability of the results.

Retrospective nature of our study is a main limitation. Another external test sample with more cases using prospective method will be required in the future to support our findings more reliably.

In conclusion, the predictive error of the Haigis-L formula is influenced by the corneal asphericity. We propose a modified Haigis-L formula based on the correlation between the prediction error and spherical aberration for 8.0 mm corneal diameter, resulting in reducing refractive errors after cataract surgery in post-corneal refractive surgery patients.
